# Identification of Chemical Bonds and Microstructure of Hydrated Tricalcium Silicate (C_3_S) by a Coupled Micro-Raman/BSE-EDS Evaluation

**DOI:** 10.3390/ma14185144

**Published:** 2021-09-08

**Authors:** Zheyu Zhu, Zhongping Wang, Yue Zhou, Yuting Chen, Kai Wu

**Affiliations:** 1 Key Laboratory of Advanced Civil Engineering Materials, Ministry of Education, Tongji University, Shanghai 201804, China; 17712937215@163.com (Z.Z.); wangzpk@tongji.edu.cn (Z.W.); 1710816@tongji.edu.cn (Y.Z.); 1930649@tongji.edu.cn (Y.C.); 2 China School of Materials Science and Engineering, Tongji University, Shanghai 201804, China

**Keywords:** coupled Raman/BSE-EDS analysis, chemical bonding, inner C-S-H, calcium hydroxide, ITZ

## Abstract

Identifying the phase evolution and revealing the chemical bonds of hydrated cements accurately is crucial to regulate the performance of cementitious materials. In this paper, a coupled Raman/BSE-EDS analysis was proposed to determine the chemical bonds of tricalcium silicate hydrates and the interface transition zone (ITZ) between inner C-S-H and anhydrates. The results show that the Raman/BSE-EDS method can accurately identify the chemical bonds of inner C-S-H and inner ITZ regions, which confirms the mixed structure of inner C-S-H and nano calcium hydroxide (CH). The inner ITZ shows a lattice change region with a thickness of 700–1000 nm, which can be attributed to the pre-disassembly process of C_3_S crystal. The successful application of coupled Raman/BSE-EDS provides new insight into the hydration process and multi-structure features of traditional cementitious materials.

## 1. Introduction

It is attractive to find solutions to tailor the performance of cementitious materials accurately in scientific and engineering society [1,2,3]. The development of nanotechnology provides the possibility of improving the macroscopic performance of concrete by regulating the hydration products at micro and even nano-level [2,3,4,5]. Tailoring material properties requires a deep understanding of the microstructure, such as the phase composition and its evolution, chemical features, morphology and etc. As the dominant mineral of Portland cement clinker, tricalcium silicate (C_3_S) reacts with water to form calcium-silicate-hydrate (C-S-H) and calcium hydroxide (CH), determining the final performance. Since this chemical reaction ensures the safe and high-efficiency service of concrete, it is necessary to enhance the understanding of the nanostructure of hydration products.

The structure characteristics of hydrated C_3_S are highly dependent on the hydration stage. The hydration of C_3_S can be divided into the following stages: (I) dissolution of C_3_S, (II) induction, (III) acceleration, (IV) deceleration, and (V) continuous stage [6]. In a typical image of C_3_S hydration process, the outer C-S-H is mainly deposited away from the edge of anhydrated particles, the inner C-S-H is formed inside the edge of original particles, and CH crystallizes in the pore solution near C_3_S [7]. Before entering the deceleration stage, the water and space are sufficient for the precipitation of outer C-S-H and CH and, finally, form a massive outer C-S-H and CH. Taylor pointed out that there are two types of CHs, i.e., crystalline CH and cryptocrystalline CH [8]. In a typical BSE image, the crystalline CH with a hexagonal plate-like stacked morphology is usually referred as a weak phase for mechanical properties. During the hydration of C_3_S, different ions are dissolved and released in the aqueous solution, and thus the structure of C-S-H and CH will change time-dependently. Moreover, the outer C-S-H and nano-CH cannot be isolated from each other. Studies indicated that the inner C-S-H and nano-CH could be mixed with each other [9,10]. This assumption is consistent with the results of coupled SEM-nanoindentation analysis [9] and high X-rays adsorption analysis [10]. However, direct experimental evidence on the local chemical features of mixed inner C-S-H and CH is still limited.

During the continuous stage, the anhydrated C_3_S is wrapped by inner C-S-H. This structure and the characteristics of the pore solution results in a rather slow hydration process. Jennings et al. [11] pointed out that the structure hinders the contact between anhydrated C_3_S and water. The slow continuous stage provides the possibility to determine the micro-nanostructure and property of hydration products (such as inner C-S-H). When the diffusion rate of water is limited, the structural transformation from C_3_S to C-S-H would proceed slowly, and a transition zone can be expected. Y. Wei [12] found that there is an interface transition zone (inner ITZ) with a lower storage modulus between the anhydrated C_3_S grain and the surrounding inner C-S-H. Because the inner ITZ is located at the boundary between anhydrated C_3_S and inner C-S-H, it is difficult to be distinguished from the surrounding minerals by traditional methods. Therefore, the nanostructure of chemical bonds of the inner C-S-H and inner ITZ is still unclear.

Recent studies have investigated the structure of C-S-H gel, CH, and anhydrated C_3_S particles, including the model of C-S-H, types of CH, and the compositions of hydrates. However, the above-mentioned structure of mixed inner C-S-H and the inner interface transition zone (inner ITZ between inner C-S-H and C_3_S particles) still need to be understood more deeply.

BSE image and Raman spectroscopy have been widely used to study cementitious materials. The BSE image can distinguish various phases and obtain the in-situ morphology [7]. Although the BSE associated with EDS can determine the chemical composition, it cannot provide information regarding the chemical bond vibration within a given region. Raman spectroscopy is usually applied to detect local structures on the nearest neighbor and second nearest neighbor scales, which is similar to the function of infrared and nuclear magnetic resonance [13,14,15,16]. However, it is still difficult to obtain an accurate local position and morphology. Defining the morphology and local spectrum structure simultaneously can provide more information on hydration products directly, especially for the mixtures or inner ITZ at nanoscale. The BSE-Raman correlative microscopy provides an ideal solution by integrating the functions of BSE image and Raman spectroscopy. It can perform a Raman spectroscopy scan on specified regions in a BSE image to obtain local structure information, morphology, and composition (combined with EDS).

In this paper, the sample preparation and determination method were investigated to evaluate the feasibility of using BSE-Raman correlative microscopy and acquire more deep information about the micro and nanostructure of hydrated C_3_S particles. The chemical bond vibration of CH in the C-S-H region and the inner ITZ between inner C-S-H and C_3_S were especially focused. The mechanism of the C_3_S hydration process during the continuous stage is thereafter discussed based on the obtained information.

## 2. Materials and Methods

### 2.1. Raw Materials

**Figure 1:**
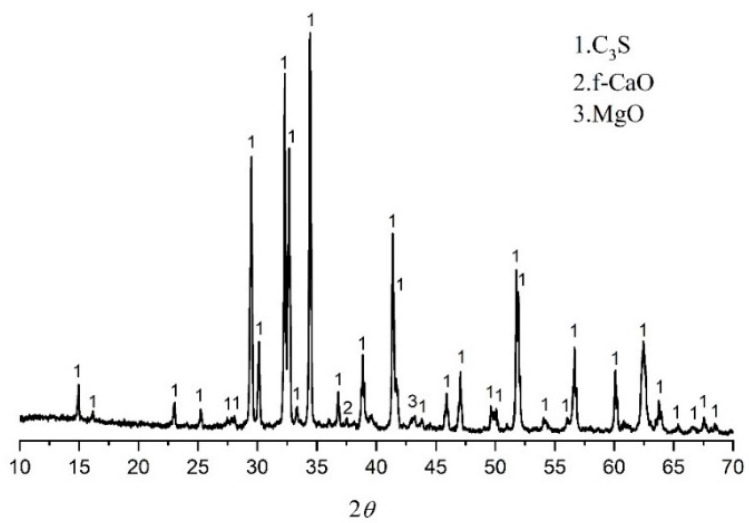
XRD of prepared M3-C_3_S.

The monoclinic (M3) type C_3_S (M3-C_3_S) was selected as the hydration sample because the structure of M3-C_3_S is similar to alite in the Portland cement clinker. The M3-C_3_S was prepared according to [17]. This can be summarized as the chemical reagent CaO. SiO_2_ was used as raw materials, and the monoclinic C_3_S structure was stabilized by MgO. The molar ratio of CaO to SiO_2_ is 3.0, and the MgO accounts for 2% of the total mass. The chemical agents were accurately weighed, mixed, homogenized, and placed in platinum crucibles, and then sintered three times in a chamber furnace at 1400 °C for 4 h. The mineralogical characteristics of C_3_S obtained in the experiment are shown in Figure 1.

### 2.2. Sample Preparation

A total of 3.00 g M3-C_3_S was mixed with 1.50 g water to prepare the hardened paste for micro and nanostructure determination. The paste was stirred, sealed, and cured at 25 °C and R.H. 95% for 28 days. After reaching the designed testing age, the hydrated paste was broken into a sheet less than 100 mm. In order to avoid the impact of organic residues on Raman analysis, ethanol was not used to terminate C_3_S hydration. Instead, the sheet was directly placed in a vacuum drying oven at 65 °C for 24 h [18].

### 2.3. BSE-EDS Analysis

The EDS area scanning was performed on the micro-region to obtain the element distribution involving calcium, silicon, and oxygen. Then, the point scan was used to evaluate the Ca/Si ratio for the selected points on inner C-S-H, outer C-S-H, and CH. The sample was not conductively coated.

### 2.4. BSE-Raman Measurement

A thin sheet of hydrated C_3_S pasted with a flat surface was selected and placed on a carbon-based conductive tape. After being placed under BSE electron microscopy and evaluated by EDS on specific areas or points, the sample was transferred to Raman microscopy and scanned by Raman imaging and point analysis. The BSE-Raman combination device ensured accurate consistency between the BSE and Raman viewing areas.

The sum Raman spectrum was obtained, and then the Raman spectral imaging was constructed with Project Five^®^ software (version 5.0, WITec, Ulm, German). The detailed construction procedure and key issues can be summarized as follows: (1) Cosmic Ray Removal (CRR) was performed to remove cosmic rays from the sum Raman spectrum. (2) The sum spectrum was smoothed, and the background was subtracted. (3) The characteristic Raman peaks were used to filter the sum Raman spectrum, and then its spatial distribution was plotted. The Raman images and BSE images were overlaid and then compared with each other. The Image Cross Section (ICS) was also analyzed with Project Five^®^ software. ICS captured the CCD counts of a specified peak intensity along a line. The Raman data of the selected point was initially performed using CRR and smoothed, and then the background signal was subtracted. The instrument of TESCAN MAIA3 GMU model 2016/WITec apyron (WITec, German) was used for BSE-Raman and EDS testing. The settings of the BSE-Raman instrument are listed in Table 1.

In order to obtain a detailed Raman spectrum, a Raman point mode was performed on the region of inner C-S-H. The detailed Raman spectrum of points was analyzed with Project Five^®^ software. Cosmic Ray Removal (CRR) was performed and then smoothed by background subtraction. The settings of laser power, number of accumulations, and integration time were 28.9 mW, 20, and 3.14 s, respectively. The BSE-Raman mapping mode can identify the hydrates with a size of at least 360 nm. If the hydrates are smaller than 360 nm, the chemical bands can be observed only in the Raman spectrum but not shown in BSE-Raman mapping. In some cases, the shape module of the Project Five software^®^ is used to provide data optimization schemes to modify the Raman spectrum by fixing the intensity of characteristic peaks and reducing the intensity of spurious peaks. It enables the Raman spectrum to highlight characteristic functional groups, while the defect of the shape module optimization could remove some detailed information in spurious Raman peaks. In order to ensure data integrity for reference, the shape module optimization was not applied in this work.

### 2.5. Construction of Raman-BSE Images

**Figure 2:**
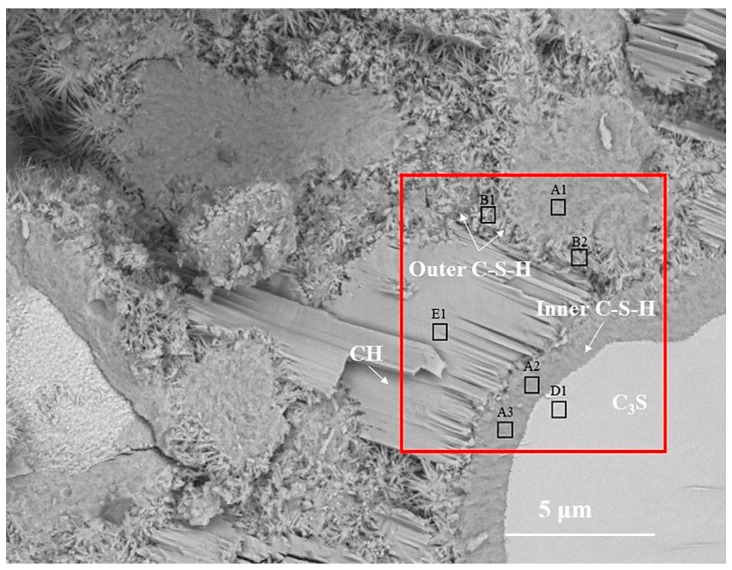
The BSE images of partially hydrated C_3_S particle after 28 d.

Figure 2 is the BSE image of C_3_S paste hydrated for 28 d. According to the gray difference and morphology, the phases such as C_3_S, inner C-S-H, outer C-S-H, and CH can be clearly distinguished. In Figure 2, the inner C-S-H is tightly integrated with anhydrated C_3_S, and the outer C-S-H is wrapped around the inner C-S-H. The CH sheets are stacked together to form a CH crystal region. The relative spatial position of hydration products and the anhydrated C_3_S is consistent with the results of [7]. The B1 region is part of the outer ITZ regions, which are usually mentioned in mortar and concrete. Once C_3_S contacts with water, Ca^2+^, H_2_SiO_4_^2−^, and OH^−^ are dissolved in water and recrystallized outside anhydrated C_3_S particles to form outer C-S-H and CH [19,20]. The formation of inner C-S-H is related to the deceleration stage [11] and the continuous stage of C_3_S hydration.

**Figure 3:**
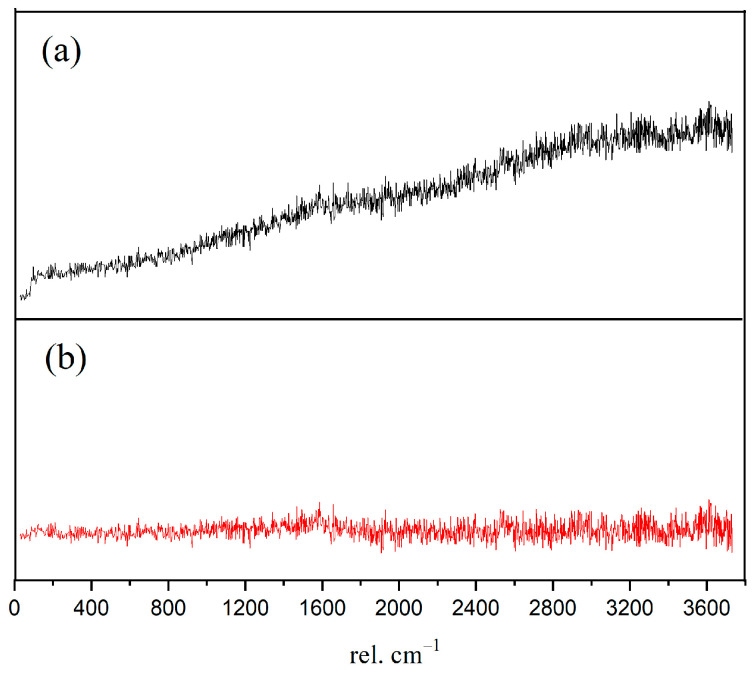
Sum Raman spectrum of the inner area within red rectangular frame: (**a**) without data processing; (**b**) after processing. Project Five^®^ software can distinguish the characteristic peaks.

In order to obtain the micro-nanostructure of the hydration products and anhydrated C_3_S in Figure 2, a red rectangular frame with a size of 10×10 μm^2^ was selected to scan the sum Raman area. Figure 3a,b is the sum Raman spectrum obtained on the inner area of the red rectangular frame (Figure 2) before and after data processing. The sum Raman spectrum is the superposition of multiple phases. The position of the hydration products can be fixed according to the distribution of characteristic peaks. The sum Raman was filtered according to the characteristic peaks in Table 2. Although the peaks seem to be weak, it is noteworthy that Project Five^®^ software can distinguish the filtered characteristic peaks in Figure 3. The characteristic vibration bands of certain minerals in the Raman spectrum can be found: 830 cm^−1^ for M3-C_3_S, 130 and 670 cm^−1^ for C-S-H, and 360 and 3620 cm^−1^ for CH [13,14,15,16]. The characteristic vibration bands of CH overlap with the broad bands of carbon [15,21]. The carbons are formed by carbon deposition in systems [21]. This phenomenon can also be found in XPS instrument [22,23]. These carbon Raman bands can be well fitted by two Gaussian distributions known as “D” and “G” bands, centered at around 1350 and 1560 cm^−1^ [24]. If the CH bands are remarkable and not completely covered by carbon bands and the broad bands are not well fitted by only those two “D” and “G” bands, the existence of CH can be confirmed indirectly. The existence of CH in the deconvolution has been discussed below. After defining the Raman spectrum, we can construct the Raman image of each phase.

**Figure 4:**
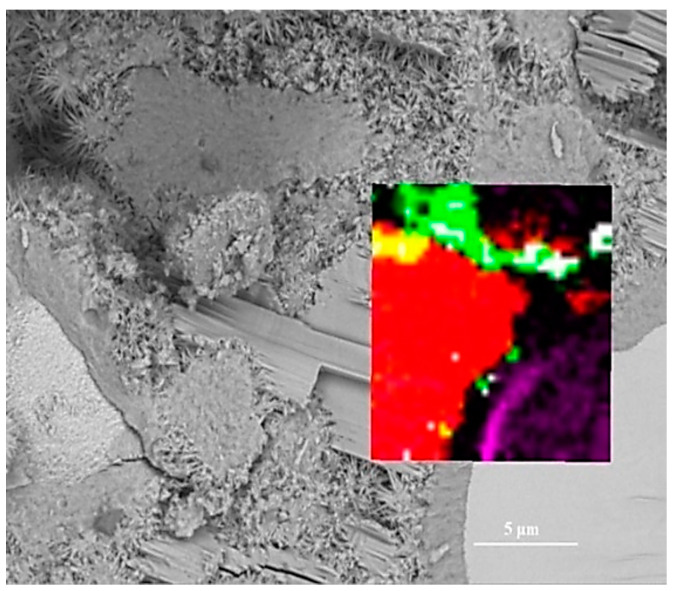
Overlay of Raman and BSE images: white for outer C-S-H; green for mixtures; red for crystalline CH; purple for C_3_S; black for inner C-S-H; yellow for mixture of outer C-S-H and crystalline CH.

**Figure 5:**
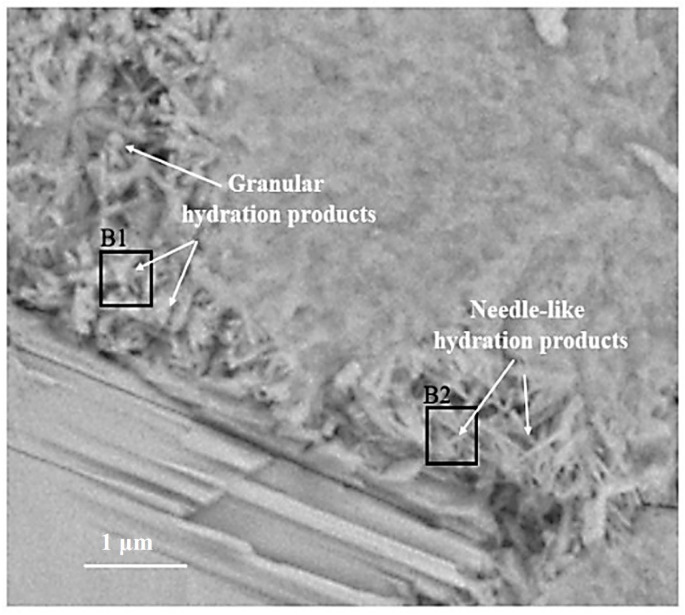
BSE image of outer C-S-H region: B1 with a Ca/Si ratio of 2.0; B2 with a Ca/Si ratio of 1.8.

To assess the positioning accuracy and obtain more detailed chemical information of observed areas, we overlapped the BSE images with Raman images. In Figure 4, the spatial position of anhydrated C_3_S and crystalline CH in the Raman imaging is consistent with the BSE observation. Filtering the sum spectrum and constructing Raman imaging can accurately identify the spatial distribution of C_3_S and crystalline CH. The outer C-S-H is located outside the hydrated C_3_S particles in the BSE image. The green region corresponds to the outer C-S-H region in the BSE image. The carbons are only distributed in the outer C-S-H region due to the high specific surface area of C-S-H, which can adsorb the deposited carbon. The unrecognized outer C-S-H causes the lack of white regions in the Raman imaging. It is due to a weaker Raman activity of C-S-H and its morphology feature. When the surface is not flat (e.g., granular morphology and substantial nano pores), the Raman laser diffuse scattering happens, resulting in the loss of local chemical bonds’ signals. In this work, the outer C-S-H is spherical and substantially porous (in Figure 5), which could lead to the scattering of diffuse in some areas and the inability to recognize outer C-S-H. The testing parameter of Raman imaging is fixed, and the laser diffuse scattering happens in some areas, resulting in recognition failure. This phenomenon cannot be avoided completely. In the BSE-Raman microscope, the BSE-EDS analysis can realize hydrates identification, spatial positioning, and supplement information provision. Then, the chemical bonds vibration bands can be obtained by adjusting the point Raman spectrum flexibly. In order to confirm the detailed chemical information of hydrates, we adjusted the test parameters to obtain the point Raman vibration spectrum. A combination of BSE and EDS was also applied to analyze the morphology and Ca/Si ratio.

## 3. Results

### 3.1. Chemical Bonds of CH in the Outer C-S-H Region

The BSE image of the outer C-S-H is shown in Figure 5. Both needle-like and granular hydration products can be observed outside the hydrated C_3_S particles. According to [7], the needle cluster in region B2 is the outer C-S-H. The formation of this structure is related to the hydration behavior of C_3_S before the continuous stage [8,25]. Some granular hydration products are distributed in region B1.

**Figure 6:**
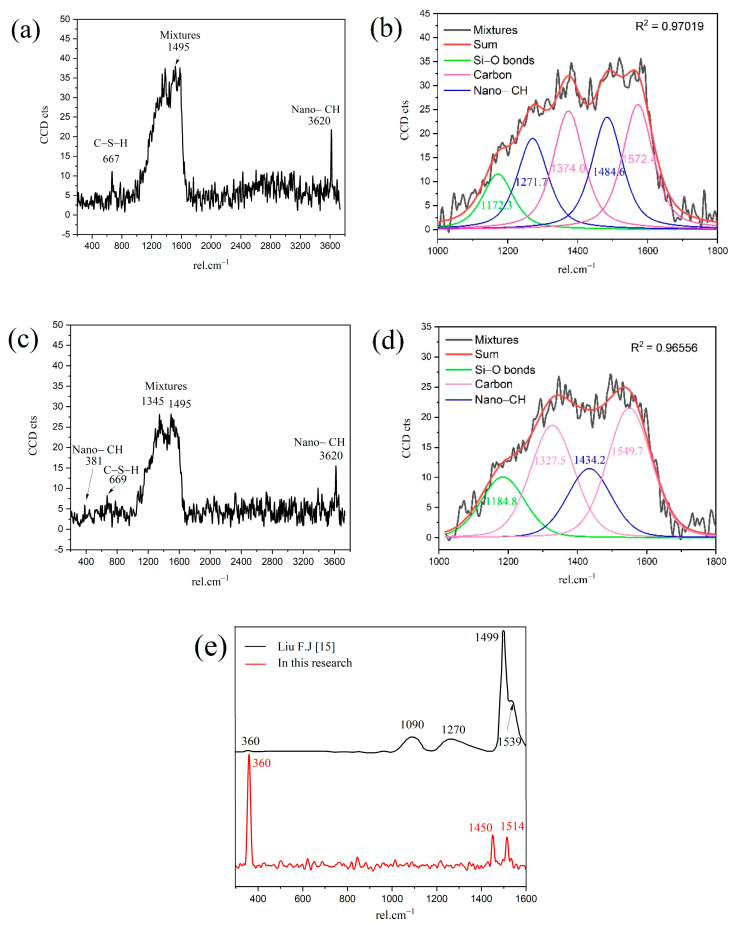
Raman spectrum of outer C-S-H region: (**a**) B1; (**b**) deconvolution of mixtures in B1; (**c**) B2; (**d**) deconvolution of mixtures in B2; (**e**) reagent CH powders.

The Ca/Si ratios of granular hydration products (region B1) and the outer C-S-H (region B2) are 2.0 and 1.8, respectively. In order to study the chemicals bonds and micro-nanostructure in the B1 and B2 region, local Raman spectroscopy was performed on the corresponding area. The Raman spectrum of the outer C-S-H is shown in Figure 6. As shown in the spectrum, the weak characteristic peaks of 667 cm^−1^ and 669 cm^−1^ correspond to C-S-H. The 3620 cm^−1^ corresponds to nano-CH. It is noteworthy that the peaks of 1200–1600 cm^−1^ in Figure 6a,c contains not only carbon vibration bands but also clear CH vibration bands (1495 cm^−1^), which can be observed in Figure 6e that the Raman spectrum of reagent CH powders has some vibration bands at about 1200–1600 cm^−1^. Thus, the characteristic vibration bands between 1200 cm^−1^ and 1600 cm^−1^ can be attributed to the mixes of nano-CH and carbons. In Figure 6b,d, the deconvolution of broad bands was performed by the Gauss–Lorentz formula. The curves can be fitted well by characteristic vibration bands of nano-CH and carbon. The fitted curves at 1327 cm^−1^, 1374 cm^−1^, 1549 cm^−1^, and 1572 cm^−1^ belong to carbon, and the fitted curves at 1271 cm^−1^ and 1484 cm^−1^ in Figure 6b and 1434 cm^−1^ in Figure 6d belong to nano-CH. The nano-CH may have a slightly different nanostructure, which results in multiple fitted peaks. If the nano-CH is small enough, the atoms on the surface affect the inner crystal structure, resulting in the crystal structure and Raman changes. Compared with C-S-H, the Raman activity of carbon is stronger, which covers up some information. In addition, the bands are not well fitted by only those two “D” and “G” bands, which also indicates that the broad bands of 1200–1600 cm^−1^ are a mixture instead of only carbon. The existence of nano-CH in the deconvolution is consistent with the BSE and EDS analysis results, which will be further discussed in the following section. In actuality, regardless of the 1200–1600 cm^−1^ Raman information, the existence of nano-CH in regions B1 and B2 can also be confirmed by 381 cm^−1^ and 3620 cm^−1^ bands. Thus, the B1 and B2 Raman characteristic peaks indicate that both regions contain outer C-S-H, CH, and carbon. Comparing Figure 6a with Figure 6c, the Raman spectra in region B2 are similar to that in region B1. This indicates that nano-CH was precipitated in both granular hydration products and the needle cluster. For C-S-H, the characteristic peak at 660 cm^−1^ is not significant in the figure. This is due to the uneven surface and the weak Raman activity of C-S-H. The atom percent of silicon in region B1 and region B2 are 9.5 and 12.1, respectively. This confirms the existence of C-S-H. In addition, region B2, with a Ca/Si ratio of 1.8, contains less CH, and region B1, with a Ca/Si ratio of 2.0, has more CH precipitation. This phenomenon can be confirmed by the formation of Ca-OH bonds in the C-S-H. M.J. Abdolhosseini Qomi [26] found that more Ca-OH bonds formed through water molecules dissociated and condensed into silica chains when the Ca/Si ratios increased from 1.1 to 2.0. This Ca-OH bond was pH dependently [27]. The other possible explanation is that outer C-S-H can grow on the surface of nano-CH particles [28].

Although the outer C-S-H distribution in the Raman imaging is consistent with the area observed by BSE, there are still regions containing the outer C-S-H that cannot be recognized by Raman imaging. In a traditional Raman microscope, those unrecognized regions can easily be ignored. In a coupled micro-Raman/BSE-EDS microscope, the unrecognized regions can be identified by a BSE image. The weaker Raman activity of C-S-H causes the failure of Raman recognition. The findings obtained through the approaches of Raman, BSE, and EDS can then be complemented by each other.

### 3.2. Chemical Bonds of CH in the Inner C-S-H Region

One of the advantages of the coupled BSE-Raman method is that when Raman imaging fails to obtain local chemical bands distribution, hydrates identification can also be performed by BSE-EDS analysis. Then, detailed chemical information can be obtained by adjusting the point Raman vibration spectrum. The characteristic vibration bands of inner C-S-H are much weaker. Filtering from the sum spectrum for inner C-S-H (black zone in Figure 4) cannot be achieved. An adjusting point Raman testing can obtain the inner C-S-H Raman spectrum. The complemented analysis can be observed by the BSE image (in Figure 2); the black area in Raman is consistent with inner C-S-H regions. This indicates that the spatial distribution of the inner C-S-H in Raman can be calibrated by subtracting the other hydration products and anhydrated C_3_S. Combining the Raman image with the BSE image, the thickness of the inner C-S-H in the BSE image is about 1 μm. Hu [10] and Chen [9] speculated a mixed structure consisting of C-S-H/ nano-CH. Chen [9] pointed out that inner C-S-H and CH are related mixtures. This speculation can be matched with micromechanical analysis through mathematical calculations. As the indication of this speculation, the Raman spectrum of inner C-S-H should contain the vibration bands of CH. In order to obtain direct evidence of the chemical bonds of these mixtures, the Raman point scan was performed.

**Figure 7:**
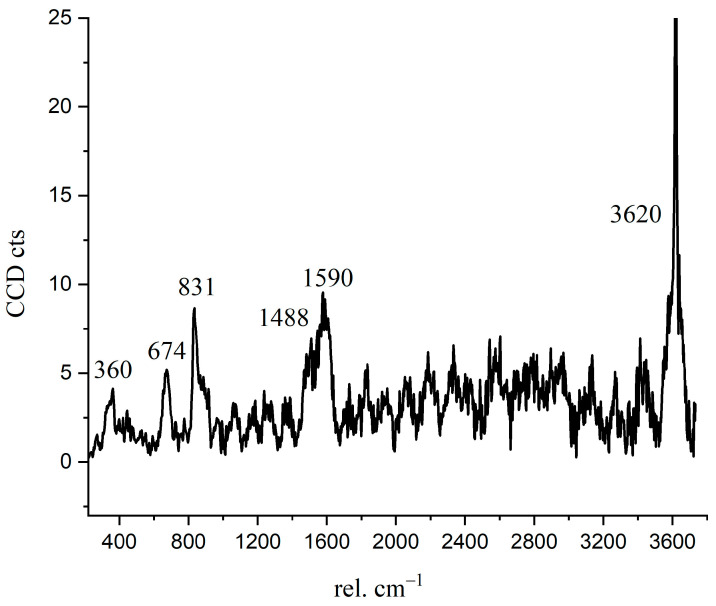
Raman spectrum of inner C-S-H region.

The Raman spectrum of the inner C-S-H is shown in Figure 7. The Raman characteristic peak of inner C-S-H is more complicated than the other hydration products. The characteristic vibration bands 674 cm^−1^ refer to C-S-H, 831 cm^−1^ refers to anhydrated C_3_S, and the peaks of 360 cm^−1^, 1488 cm^−1^, 1590 cm^−1^, and 3620 cm^−1^ correspond to nano-CH. The coexistence of CH in the inner C-S-H was directly confirmed in this work by spectroscopic evidence. This indicates that the inner C-S-H mixed with CH as a composite. Another interesting observation is the presence of the chemical bonds of anhydrated C_3_S in the inner C-S-H region. As shown in Table 3, the Ca/Si ratios of inner C-S-H were 1.8, 2.1, and 2.1. Allen [29] detected that the Ca/Si ratio for C-S-H was 1.7 by combining small-angle neutron and X-ray scattering data. The classical Ca/Si ratio was 0.7–2.0 in a synthesized C-S-H [11,26,29,30]. The Ca/Si ratios of inner C-S-H at A2 and A3 are greater than the above-mentioned values. This may be due to the coexistence of C-S-H/ nano-CH composites. The high Ca/Si ratios of inner C-S-H obtained by EDS can be attributed to the mixture of C-S-H, CH, and anhydrated C_3_S. Therefore, the combination of BSE and Raman spectrum is able to provide more direct evidence of a hydrated system, which is essential for enhancing the understanding of cement hydration.

Compared with outer C-S-H, the inner C-S-H has a lack of space and water and shows a denser microstructure. The hydration process of unreacted C_3_S under this condition is different from that having sufficient moisture and space. However, both the outer C-S-H and inner C-S-H contain the chemical bond vibration of CH. According to the literature, one possible structure based on the Jennings colloidal model-II can be used to explain the phenomenon [29,30]. As described in this model, C-S-H was considered to be a clustered nanostructure packed by layered units and appears as zeolite-like cavities. Those cavities provide space for physisorbed water molecules [30]. Long silicate chains of C-S-H could be decomposed into shorter ones, and new defect regions formed as the Ca/Si ratio increased from 1.3 to 2.0. If the CH was small enough, the defect regions and zeolite-like cavities could also be filled with CH. The other possible structure is based on the low-density (LD) and high-density (HD) C-S-H model proposed by Jennings [31]. In this model, C-S-H is composed of nano-sized units and high-density C-S-H with 24% porosity and low-density C-S-H with 37% porosity. Therefore, the nano-CH may fill the pores that formed both in and out of the C-S-H gel at the molecular scale.

### 3.3. Chemical Bonds in the Crystalline CH

**Figure 8:**
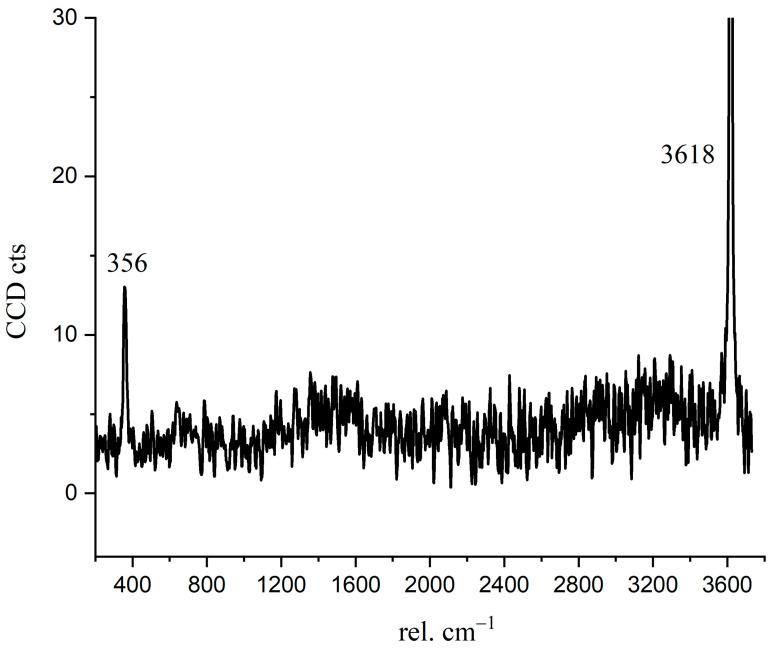
The Raman spectrum of crystalline CH.

Crystalline CH is usually plate-like [32], which is difficult to have a skeleton effect as the needle-like ettringite. The interface fragility and the layered cleavage surface result in poor mechanical properties of concrete [33]. To confirm the Raman spectrum of crystalline CH formed during C_3_S hydration, we detected and analyzed the Raman spectrum of hexagonal CH observed in the BSE image. In Figure 8, the characteristic peaks of crystalline CH were 356 cm^−1^ and 3618 cm^−1^. As confirmed in Figure 4, the distribution of 356 cm^−1^ and 3618 cm^−1^ peaks correspond to the crystalline CH observed in the BSE image (red region). For reagent grade CH, the Raman characteristic peaks were 360 cm^−1^, 3620 cm^−1^, and 1200–1600 cm^−1^ [15,34]. Liu [15] described that the broad peaks of 1200–1600 cm^−1^ correspond to cryptocrystalline CH. In this research, the peaks of 1200–1600 cm^−1^ in Figure 6 not only indicate the formation of nano-CH but also the presence of carbon. The fitted curves (in Figure 6) at 1271 cm^−1^, 1484 cm^−1^, and 1434 cm^−1^ partially belong to cryptocrystalline CH. When C_3_S particles are in contact with water, the solution is abundant in Ca^2+^ and OH^−^; thus, the formed CH types may be diverse. Tylor pointed out that cryptocrystalline CH can be found in hydrated cement, pastes made from calcium oxide and silica with a great ratio of water to solid, and also in the alkali-activated hydration products of blast furnace slag [8]. The structure of cryptocrystalline CH shows that the surface of Ca^2+^ in the cement paste was absorbed by OH^−^ [8]. Nshan Zulumyan [35] reported that CH would react with Si-O-H silanol groups on the surface of the granular silica during stirring, resulting in the formation of calcium hydroxosilicate units, such as ≡Si-O-Ca-OH.

### 3.4. The Inner ITZ between Inner C-S-H and C_3_S

Since the anhydrated C_3_S particles are wrapped by inner C-S-H, the hydration will be slowed down prominently. Therefore, the structure variation of C_3_S crystal at the micro-nanoscale during the hydration process can be obtained by Raman analysis. The brightness of the constructed Raman images was used to determine the concentration of the substance and the crystal structure. The stronger the Rayleigh scattering signal, the brighter the phase of the related Raman band. By comparing the brightness of the same substance, the relative content and the variation of crystals can be evaluated.

**Figure 9:**
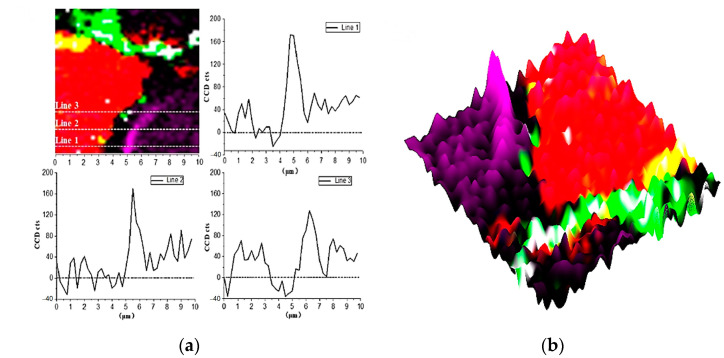
Raman imaging of 830 cm^−1^ peaks distribution: (**a**) cross-section linear analysis; (**b**) the intensity of 830 cm^−1^ characteristic peaks.

**Figure 10:**
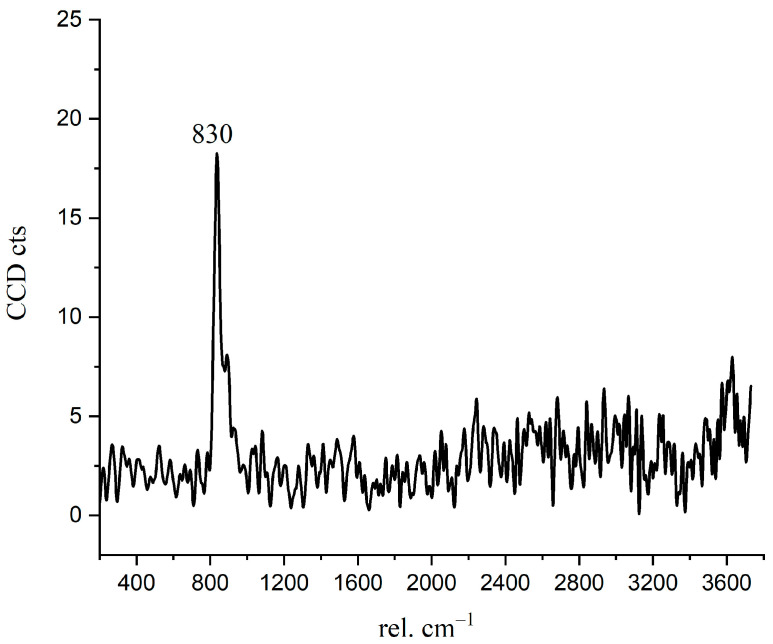
The Raman spectrum of inner ITZ region.

In Figure 4 and Figure 9b, there is a bright purple region distributed along the boundary between the inner C-S-H and C_3_S phases. Wei [12] found that the ITZ shows the lowest storage modulus in comparison with the inner C-S-H and anhydrated C_3_S. Figure 10 is the Raman spectrum of the ITZ region; the characteristic peak is only observed at 830 cm^−1^. This peak represents the Si-O bonds of the ν_1_ silica tetrahedron vibration in the C_3_S crystal [6]. No characteristic peaks of C-S-H and CH were found. This indicates that the ITZ possesses the same chemical bonds of silica tetrahedron as anhydrated C_3_S. 

In order to obtain more detailed information on ITZ, as shown in Figure 9a, the Image Cross Section (ICS) was performed on the intensity change of 830 cm^−1^ in Raman imaging. Along all the three lines in Figure 9a, the intensity of the 830 cm^−1^ peak near the edge of inner C-S-H is about 2–2.5 times greater than that of inside C_3_S. In addition, the enhanced width of the characteristic peak along the straight line is about 700–1000 nm, which indicates the thickness of the inner ITZ. The signal of the characteristic peak at 830 cm^−1^ is stronger in the bright purple region. On the one hand, it may be caused by the lattice change of C_3_S. The intensity of the Raman peak is proportional to the change in the dipole moment of the induced transition. The increased distance of the atoms causes the polarizability enhancement. On the other hand, it could be due to an increase in the concentration of the substance. The 830 cm^−1^ represents the vibration of the silica tetrahedron in C_3_S. The enrichment of the silicon tetrahedron can cause an increase in the intensity Raman spectrum.

**Figure 11:**
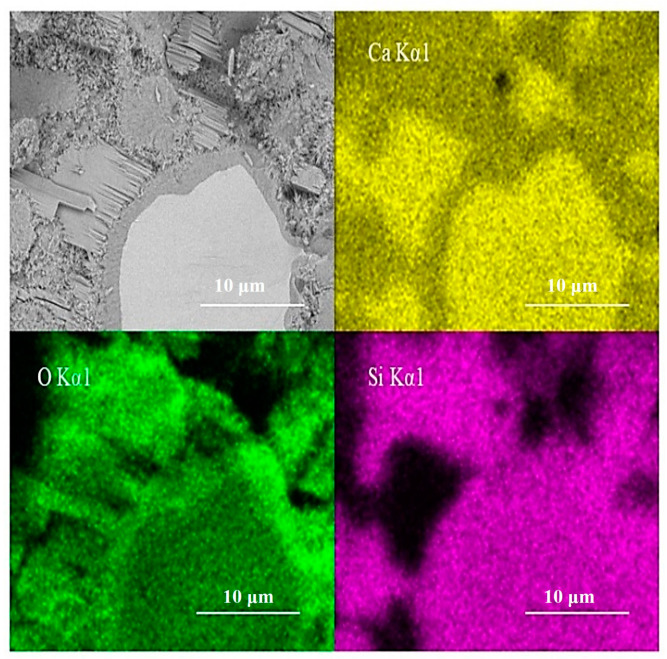
Elements concentration of EDS scanning analysis.

EDS scanning of the test area is shown in Figure 11. The inner C-S-H region shows less calcium and similar silicon content in comparison with anhydrated C_3_S. The silicon content does not increase along the boundary between the inner C-S-H and anhydrated C_3_S particles. This is consistent with the finding of Y. Sakalli [36]. However, it cannot exclude silicon enrichment completely. The doubt is that the emission volume of EDS is significantly bigger than the discussed inner ITZ width. In actuality, it is difficult to judge the accurate width of the emission volume of EDS. Thus, both crystal lattice changes and silicon enrichment have the possibility to enhance the intensity of the 830 cm^−1^ peak.

### 3.5. Discussion

#### 3.5.1. The Formation of Inner ITZ between C-S-H and Anhydrates

Both crystal lattice changes and enriched silicon have the possibility to cause the formation of inner ITZ between C-S-H and anhydrates. This can be explained by the hydration process of C_3_S. Bullard [11] pointed out that water and space have a great impact on the hydration process of C_3_S. Some scholars believed [11,37,38] that when moisture and space are sufficient, C_3_S will rapidly dissolve after wetting according to the following chemical reaction: C_3_S + 3H_2_O→Ca^2+^ + H_2_SiO_4_^2−^ + 4OH^−^. This determines the stages of (I) dissolution of C_3_S, (II) induction, and (III) acceleration. Hu [10] and Scrivener [7] described that in the continuous stage, the inner C-S-H is uniformly wrapped around anhydrated C_3_S particles, and external water is insulated from the anhydrated particles. This leads to a lack of space and moisture in the internal hydration environment during the continuous stage. Therefore, the hydration of C_3_S is highly limited and mainly controlled by water molecules and the diffusion rate of Ca^2+^.

What of the lattice changes gradually formed in the disintegration of C_3_S? The initial reaction mechanism of crystals dissolved in water has attracted widespread attention. Theoretically, the dissolution of crystals in water is related to the cationic dissolution and formation of hydrated cation ions [39]. For example, J. Peng [39] observed that Na^+^ reacted quickly with H_2_O to form hydrated Na^+^ ions when NaCl crystals were dissolved. In addition, he also pointed out that Na^+^ hydrated with water and diffused more quickly than other ion hydrates [39]. Since NaCl and C_3_S are both ionic crystals, the dissolution of C_3_S also has the formation stage of hydrated cation ions. The Ca^2+^ hydrates (Ca^2^^+^ nH_2_O) are formed firstly by hydrogen bond when the diffused water molecules contact with calcium ions. Once the space is limited, the migration of Ca^2+^ hydrates would be hindered. Then, Ca^2+^ hydrates can only be precipitated in the vicinity of the crystal lattice. In addition, this results in an increased volume and polarizability of the nearby C_3_S crystal and a stronger Raman peak. Based on the conjecture of the formation of Ca^2+^ hydrates, the hydration number of calcium ions may affect the structure of C-S-H. Coincidentally, the distances between the Ca-O bonds of 2.39 Å in the C-S-H structure is close to that of 2.40 Å in the Ca^2+^ hydrates with a 7 hydration number [40,41]. The details of this speculation need to be further confirmed, in which DFT calculations and MD simulations are potential methods.

What of the silicon enrichment in the inner ITZ region? As discussed above, the microstructure of hydrated C_3_S in the continuous stage is similar to eggs, in which the dense inner C-S-H play as “shells” wrapping the anhydrated C_3_S particles. One possibility is these dense inner C-S-H “shells” may hinder water migration, which causes a slow hydration rate. Theoretically, the migration of ions is dominated by the migration of water molecules. When parts of calcium ions migrate out from the lattice, the silica tetrahedron, which loses calcium ions, will migrate gradually. Given the hindering effects of dense inner C-S-H “shells,” silica tetrahedron may aggregate between C_3_S and inner C-S-H regions. However, this silicon enrichment region has not been observed by scholars. Thus, it is reasonable to speculate that the silicon enrichment region is so thin enough that it is out of the detection limit of the instrument, or the silicon enrichment region does not exist. Distinguishing different crystal lattices, their orientation, or excluding silicon enrichment can be measured by EBSD or over-contrasted BSE imaging.

#### 3.5.2. Supplement for the Microstructure of Hydrated C_3_S

Recent research has provided the C-S-H model from the mesoscale to nanoscale. Scrivener [7] proposed that according to the BSE images, C-S-H can be classified into an inner C-S-H and outer C-S-H. Another way to classify C-S-H has been described; with the help of advanced nano-indentation [9,42], transmission electron microscopy (TEM) [43], and small-angle neutron scattering (SANS) [29,44], C-S-H can be classified into low-density C-S-H (LD C-S-H) and high-density C-S-H (HD C-S-H). These roughly correspond to the outer C-S-H and inner C-S-H, as defined in the BSE image [19,45,46]. In previous studies on the nanoscale of the C-S-H structure, two classic models for the C-S-H structure were established. Taylor [29,45] pointed out that the C-S-H gel was the defective 1.4 tobermorite or jennite, in which the SiO_4_ tetrahedra chains of tobermorite or jennite were fractured. In addition, the defective SiO_4_ tetrahedra chains resulted in a broad Ca/Si ratio ranging from 0.83 to 2.25. Another model proposed by Richardson [47] was the tobermorite/jennite and tobermorite/CH model, in which the mixture of CH and C-S-H that matched the Ca/Si ratio was obtained by EDS. This paper supplements some results for the microstructure of hydrated C_3_S. The BSE/Raman-EDS results in this work confirmed the mixed structure at nanoscale. The tobermorite/CH model is close to the real structure of hydrated C_3_S. The analysis shows that the chemical bond provided an important indicator for this mixed structure. The spatial distribution of hydrated C_3_S can be proposed through the BSE/Raman-EDS analysis. A mixed nano-CH and C-S-H model inspired by the chemical bonds was obtained. The nano-sized CH is distributed in the inner C-S-H region. There is a lattice change layer between C_3_S and inner C-S-H, and its thickness is about 700–1000 nm.

The aggregate is considered to be the major component of the cementitious material. When aggregates incorporate into the mixtures, the Ca/Si molar ratios are various at different ITZ regions. The nanostructure of the C-S-H/CH mixtures is also changed with the local Ca/Si molar ratios, such as C-S-H unit types and the ratios of C-S-H to CH. The effect of the Ca/Si ratios on the strength of the cementitious material and synthetic C-S-H have been researched by nano-indentation. Recent research [42,48] has demonstrated that Young’s modulus ranges from 21 to 50 GPa, and the elastic modulus and hardness increased when the Ca/Si molar ratio of C-S-H decreased. Thus, lower Ca/Si ratios may benefit a better-strength cementitious material. It is still worth mentioning that the result is based on a limited number of experiments. Thus, it needs to be further validated with more extended experiments and other potential methods. For example, AFM-IR and TERS [49,50,51] could be a useful method, and molecular dynamics simulation (MS) will provide more detailed structure compositions. In addition, the microstructure of hardened cement paste is highly dependent on the cement type [52], service environment [53,54], such as blended cement [55], and clinker composition [56]—the results would need to be further evaluated.

## 4. Conclusions

BSE-Raman correlative microscopy combined with Raman/BSE-EDS is a powerful method that can reveal the local micro-nanostructure, chemical bonds, and compositions of hydrated cementitious materials. In this paper, the hydration products and inner ITZ of C_3_S were evaluated by BSE-Raman correlative microscopy. In addition, the spatial distribution model of the hydrated C_3_S structure was established.

The spectroscopic determination indicated that the inner C-S-H is actually a mixture of C-S-H and CH. The lattice change of the C3S crystal with a thickness of 700–1000 nm was found along the inner ITZ between inner C-S-H and anhydrated C_3_S. There is a pre-disassembly process of C_3_S crystal characterized by enhanced Raman characteristic peaks at 830 cm^−1^. The formation of Ca^2+^ hydrates and lack of space may cause the aggregation of silicon elements and the crystal lattice to change.

According to the characteristic peaks of outer C-S-H, CH, and C_3_S, the sum Raman spectrum was filtered, and the Raman imaging was constructed. The spatial distributions of the hydration products of the Raman image were consistent with that of BSE. The characteristic peaks of plate-like crystalline CH were 356 cm^−1^ and 3618 cm^−1^. The successful application of micro-Raman/BSE-EDS in determining the multi-structure of hydrated C_3_S provides an important method for cementitious materials investigation and tailoring.

## Figures and Tables

**Table 1:** Instrument settings for BSE, Raman imaging, and EDS.

BSE	Acceleration Voltage	5 kV
Raman imaging	Points per line	40
	Lines per image	40
	Scan speed	40.00 s/Line
	Retrace speed	1.00 s/Line
	Integration time	1 s
	Excitation wavelength	532 nm
	Laser power	29.19 mW
	Spot size	360 nm
EDS	Live time (acquisition time)	84 s
	Acceleration voltage	15 kV

**Table 2:** Characteristic peaks for deconvolution of the sum Raman spectrum.

Region in Figure 2	Phase	Chemical Compounds	Characteristic Peaks/cm^−1^	Colors in Figure 4
Outer C-S-H	C-S-H	(CaO)_n_·(SiO_2_)·yH_2_O	130	White
			667	
	Mixtures	-	1200–1600	Green
CH	Crystalline CH	Ca(OH)_2_	356	Red
			3620	
C_3_S	C_3_S	3CaO·SiO_2_	830	Purple
Inner C-S-H	Inner C-S-H	(CaO)_n_·(SiO_2_)·yH_2_O	-	Black

**Table 3:** EDS results of the various regions in Figure 2.

Phase	Position	Ca/(Atom%)	Si/(Atom%)	Ca/Si
Inner C-S-H	A1	17.0	9.2	1.8
	A2	21.1	9.9	2.1
	A3	20.9	9.9	2.1
Mixture region of nano-CH and outer C-S-H	B1	18.9	9.5	2.0
	B2	21.7	12.1	1.8
C_3_S	D1	27.8	9.5	2.9
Crystalline CH	E1	36.9	0.5	-

## Data Availability

The data presented in this study are available on request from the corresponding author.
